# Analysis of Race and Sex Bias in the Autism Diagnostic Observation Schedule (ADOS-2)

**DOI:** 10.1001/jamanetworkopen.2022.9498

**Published:** 2022-04-26

**Authors:** Luther G. Kalb, Vini Singh, Ji Su Hong, Calliope Holingue, Natasha N. Ludwig, Danika Pfeiffer, Rachel Reetzke, Alden L. Gross, Rebecca Landa

**Affiliations:** 1 Center for Autism and Related Disorders, Kennedy Krieger Institute, Baltimore, Maryland; 2 Department of Mental Health, Johns Hopkins Bloomberg School of Public Health, Baltimore, Maryland; 3 Department of Neuropsychology, Kennedy Krieger Institute, Baltimore, Maryland; 4 Department of Psychiatry and Behavioral Sciences, Johns Hopkins University School of Medicine, Baltimore, Maryland; 5 Department of Epidemiology, Johns Hopkins Bloomberg School of Public Health, Baltimore, Maryland; 6 Center on Aging and Health, Johns Hopkins Bloomberg School of Public Health, Baltimore, Maryland

## Abstract

**Importance:**

There are long-standing disparities in the prevalence of autism spectrum disorder (ASD) across race and sex. Surprisingly, few studies have examined whether these disparities arise partially out of systematic biases in the Autism Diagnostic Observation Schedule, Second Edition (ADOS-2), the reference standard measure of ASD.

**Objective:**

To examine differential item functioning (DIF) of ADOS-2 items across sex and race.

**Design, Setting, and Participants:**

This is a cross-sectional study of children who were evaluated for ASD between 2014 and 2020 at a specialty outpatient clinic located in the Mid-Atlantic region of the US. Data were analyzed from July 2021 to February 2022.

**Exposures:**

Child race (Black/African American vs White) and sex (female vs male).

**Main Outcomes and Measures:**

Item-level biases across ADOS-2 harmonized algorithm items, including social affect (SA; 10 items) and repetitive/restricted behaviors (RRBs; 4 items), were evaluated across 3 modules. Measurement bias was identified by examining DIF and differential test functioning (DTF), within a graded response, item response theory framework. Statistical significance was determined by a likelihood ratio χ^2^ test, and a series of metrics was used to examine the magnitude of DIF and DTF.

**Results:**

A total of 6269 children (mean [SD] age, 6.77 [3.27] years; 1619 Black/African American [25.9%], 3151 White [50.3%], and 4970 male [79.4%]), were included in this study. Overall, 16 of 140 ADOS-2 diagnostic items (11%) had a significant DIF. For race, 8 items had a significant DIF, 6 of which involved SA. No single item showed DIF consistently across all modules. Most items with DIF had greater difficulty and poorer discrimination in Black/African American children compared with White children. For sex, 5 items showed significant DIF. DIF was split across SA and RRB. However, hand mannerisms evidenced DIF across all 5 algorithms, with generally greater difficulty. The magnitude of DIF was only moderate to large for 2 items: hand mannerisms (among female children) and repetitive interests (among Black/African American children). The overall estimated effect of DIF on total DTF was not large.

**Conclusions and Relevance:**

These findings suggest that the ADOS-2 does not have widespread systematic measurement bias across race or sex. However, the findings raise some concerns around underdetection that warrant further research.

## Key Points

**Question:**

Is a reference standard measure of autism spectrum disorder (ASD), the Autism Diagnostic Observation Schedule, Second Edition (ADOS-2), systematically biased across sex and race?

**Findings:**

In this cross-sectional study of 6269 children evaluated at an ASD specialty clinic in the US, 11% of ADOS-2 diagnostic items demonstrated bias for Black/African American vs White children and for female vs male children. The magnitude of bias was moderate to large for only 2 repetitive/restricted behavior interest items.

**Meaning:**

Although the ADOS-2 demonstrated minimal bias overall, these findings suggest that further research is needed to address some evidence of underdetection of ASD symptoms in Black/African American children and female children.

## Teaser

This cross-sectional study examines differential item functioning of the Autism Diagnostic Observation Schedule, Second Edition, across sex and race.

## Introduction

Autism spectrum disorder (ASD) is characterized by deficits in social communication and the presence of restricted and repetitive behaviors (RRBs).^1^ With an early onset,^2,3^ high heritability,^4^ and increasing prevalence (now 1 in 44 children),^5^ ASD is one of the most common neurodevelopmental disorders. Disparities in the prevalence of ASD by sex is one of the most consistently replicated findings, with male children being 4 times more likely than female children to receive a diagnosis.^5^ Despite a longstanding history of underdetection of ASD in minoritized racial and ethnic groups, the Centers for Disease Control and Prevention has reported no difference in prevalence estimates between Black/African American and non-Hispanic White 8-year-old children since 2016.^5^

Underidentification and delayed diagnosis of ASD has been consistently reported in minoritized racial groups,^5,6,7^ leading to disparities in access to interventions. For instance, Black/African American children are less likely than non-Hispanic White children to have an evaluation by age 3 years.^5^ On average, Black/African American children with intellectual disability receive a diagnosis 6 months later than non-Hispanic White children with intellectual disability.^5^ There are many mechanisms associated with such disparities, including lack of access to care, stigma, implicit and explicit clinician biases, and developmental literacy.^7,8,9,10,11,12,13,14^ Indeed, standardized diagnostic assessments used to inform diagnosis may also contribute to disparities in the timing and accuracy of an ASD diagnosis across sex and racial groups.^15,16^

The Autism Diagnostic Observation Schedule, Second Edition (ADOS-2),^17^ has been widely used for aiding in clinical diagnosis of ASD and is now regarded as the reference standard assessment for ASD.^18,19^ The ADOS-2 is a standardized, semistructured observational measure of ASD symptoms, providing specific probes for evaluating communication, social interaction, play, and RRBs.^17^ There have been multiple studies demonstrating the clinical utility and accuracy of the ADOS-2 across national and international samples.^20,21,22,23,24,25,26,27,28^ However, to our knowledge, there have only been 2 studies examining ADOS measurement bias at the item level, using item response theory (IRT), by sex and/or race. Specifically, Harrison et al^16^ investigated the role of race, ethnicity, and sex on 10 items of the ADOS-Generic. No measurement bias was found by sex, and a small but significant item-level bias was found for Black/African American children on 3 ADOS-Generic items. Although the findings suggest that these items may result in overestimation of impairment for Black/African American children, the sample size for this group was quite small (95 children), and the version of the ADOS used is now outdated. Second, Ronkin et al^29^ examined sex differences in social communication, between boys and girls, using the ADOS-2 Toddler version. Their results did not reveal any differences across groups.

The current study examines whether the ADOS-2 systematically underestimates ASD severity at the item level, by race (Black/African American vs White children) or sex (female vs male children), in a large clinical sample of children evaluated for ASD. We hypothesize that no substantive item-level biases will exist in the ADOS-2, given that no study has established significant item-level biases of the ADOS-2 using modern measurement methods. This study fills a critical gap in the literature considering that, to our knowledge, no studies have investigated item-level measurement bias of the most recent version of the ADOS (ie, ADOS-2), beyond the ADOS-Toddler, by race or sex.

## Methods

### Setting

Data for this cross-sectional observational study were obtained from children evaluated for ASD at an urban, outpatient ASD specialty clinic located in the Mid-Atlantic region of the US between 2014 and 2020. The clinic provides a wide range of ASD-specific medical, therapeutic, and diagnostic and treatment services. Referrals to the clinic come from a variety of sources (eg, pediatricians or parent-initiated), most of which (83%) are from within the state.

All data for this study came from the children’s electronic medical records. To be included in the analytical sample, children must have been younger than 18 years and received an ADOS-2 module 1, 2, or 3 assessment during their clinical evaluation. Children with a reported Hispanic ethnicity were excluded from the racial analysis only. This study was approved by the Johns Hopkins Medical Institutional Review Board. This study was conducted under a waiver of consent, granted by the governing institutional review board, because it used retrospective, deidentified data from the electronic medical record. This study follows the Strengthening the Reporting of Observational Studies in Epidemiology (STROBE) reporting guideline.

### Measures

#### Demographic Data

Demographic data included child’s age, insurance type, child’s race and ethnicity, and sex. Child age reflected the age at ADOS-2 administration. Insurance type was classified as public (reflecting Medical Assistance) vs private (eg, preferred provider organization) plans. Race, as reported by parents and documented in the medical records, was categorized as a 4-level variable (White, Black/African American, Asian, and other, which included Native American, Pacific Islander, multiracial, and any other race). Unfortunately, ethnicity was reported as a racial category before 2019. This resulted in the inability of informants to report both race and ethnicity during most of the study period (see the Limitations section later for details).

#### Autism Diagnostic Observation Schedule, Second Edition

The ADOS-2 is a reference standard, semistructured observational assessment used to evaluate the presence or absence of ASD-related symptoms.^17^ Only modules 1, 2, and 3 are included in this study because of sample size limitations in modules T (toddler version) and 4 (verbal, adolescent or adults). Items were harmonized across modules to ensure that each item was measuring similar content. To accomplish this, we built upon the widely accepted 2-factor framework developed by Gotham et al.^30^ This algorithm ensures content equivalence across developmental groups defined by ADOS-2 modules and algorithms. The 2-factor framework included 2 constructs, social affect (SA) and RRB subscales, that were measured using 10 and 4 items, respectively. Modules 1 and 2 have 2 algorithms based on the child’s language ability and age (≤5 years), respectively. As such, a total of 5 harmonized algorithms were used (module 1, No Words [1.1]; module 1, Some Words [1.2]; module 2, Young [2.1]; module 2, Old [2.2]; module 3). No child had more than 1 ADOS per algorithm.

The ADOS-2 was administered by a licensed clinician, including psychologists (33%) and speech-language pathologists (67%), as part of a diagnostic evaluation. Clinicians who administered the ADOS-2 completed a clinical training workshop with a certified ADOS-2 trainer. Clinicians received quarterly booster trainings that were led by a research-reliable, doctoral-level psychologist. The trainer monitored ADOS-2 reliability, and the trainee had access to other research-reliable ADOS-2 trainers for consultation. Although ADOS-2 fidelity was routinely monitored, not all the clinicians in this study reached research reliable status. Thus, the findings reflect actual clinical practice.

#### ADOS-2 Classification and Severity

ADOS-2 classification, as reported in Table 1, was determined by established ADOS-2 cutoffs for autism and ASD.^17,24^ In our clinic, these cutoffs have sensitivity of 97% and specificity of 71% for diagnosis (5353 patients). ASD severity was measured using the ADOS-2 Calibrated Severity Score. The Calibrated Severity Score facilitates comparisons across modules.^31,32^ The score ranges from 1 to 10, with higher scores reflecting greater ASD severity.^31,32^

**Table 1.** Sample Characteristics Across ADOS-2 Module Algorithms

Characteristic	Children, No. (%)					
	Total (N = 6263)	Module 1, algorithm 1 (n = 808)	Module 1, algorithm 2 (n = 1039)	Module 2, algorithm 1 (n = 828)	Module 2, algorithm 2 (n = 582)	Module 3 (n = 3006)
ADOS-2 Calibrated Severity Score, mean (SD)	5.41 (2.90)	6.76 (2.31)	6.19 (2.66)	5.14 (2.81)	5.60 (2.65)	4.81 (3.01)
ADOS-2 status						
No	2033 (32.5)	80 (9.90)	198 (19.1)	270 (32.6)	169 (29.1)	1316 (43.9)
Autism spectrum disorder	743 (11.9)	90 (11.1)	144 (13.9)	135 (16.3)	36 (6.20)	338 (11.3)
Autism	3479 (55.6)	638 (79.0)	697 (67.1)	422 (51.0)	376 (64.7)	1346 (44.9)
Practitioner type						
Psychologist	2103 (33.6)	120 (14.9)	196 (18.9)	267 (32.2)	197 (33.8)	1323 (44.0)
Speech language pathologist	4160 (66.4)	688 (85.1)	843 (81.1)	561 (67.8)	385 (66.2)	1683 (56.0)
Race						
Asian	1096 (17.5)	154 (19.1)	226 (21.8)	143 (17.3)	131 (22.5)	442 (14.7)
Black/African American	1619 (25.9)	289 (35.8)	286 (27.5)	177 (21.4)	207 (35.6)	660 (22.0)
White	3151 (50.3)	289 (35.8)	426 (41.0)	443 (53.5)	211 (36.3)	1782 (59.3)
Other^a^	397 (6.34)	76 (9.41)	101 (9.72)	65 (7.85)	33 (5.67)	122 (4.06)
Ethnicity						
Hispanic	546 (8.72)	93 (11.5)	119 (11.5)	54 (6.52)	75 (12.9)	205 (6.82)
Hispanic not reported	5714 (91.3)	715 (88.5)	919 (88.5)	774 (93.5)	507 (87.1)	2799 (93.2)
Insurance						
Public	2547 (40.8)	396 (49.2)	441 (42.6)	271 (32.9)	263 (45.4)	1176 (39.3)
Private	99 (1.59)	20 (2.48)	12 (1.16)	10 (1.22)	14 (2.42)	43 (1.44)
Other	3591 (57.6)	389 (48.3)	581 (56.2)	542 (65.9)	302 (52.2)	1777 (59.3)
Sex						
Female	1293 (20.6)	184 (22.8)	198 (19.1)	189 (22.8)	110 (18.9)	612 (20.4)
Male	4970 (79.4)	624 (77.2)	841 (80.9)	639 (77.2)	472 (81.1)	2394 (79.6)
Age, mean (SD), y	6.77 (3.27)	4.14 (2.00)	3.97 (1.52)	4.18 (0.64)	6.73 (2.00)	9.16 (2.71)
Location						
Within city limits	1487 (23.7)	242 (30.0)	285 (27.4)	200 (24.2)	167 (28.7)	593 (19.7)
Within state limits	3698 (59.0)	437 (54.1)	575 (55.3)	439 (53.0)	299 (51.4)	1948 (64.8)
Outside state	1078 (17.2)	129 (16.0)	179 (17.2)	189 (22.8)	116 (19.9)	465 (15.5)

Abbreviation: ADOS-2, Autism Diagnostic Observation Schedule, Second Edition.

^a^ Other includes Native American, Pacific Islander, multiracial, and any other race.

### Statistical Analysis

#### Item Response Theory

IRT is a method for item and test evaluation.^33^ As opposed to classical test theory, the focus of analysis in IRT models is the item and not the test or individual. IRT assumes that performance on a test item reflects an individual’s overall ability (or ASD severity in this study) on a latent trait. The IRT framework used in this study was the graded response model, a multicategory extension of the 2-parameter logistic model.^34^ The parameters calculated included item difficulty (*b_i_*) for each category of response and overall item discrimination (*a_i_*). Item difficulty is a location parameter that reflects the probability of response on the basis of an observation’s level on the latent trait (θ). Thus, higher values of *b_i_* imply that a higher level of ASD (as measured by θ) is needed to endorse the response. Discrimination measures the degree to which an item distinguishes between groups (in this study, children with or without ASD). For the IRT-based analyses, all items scores with a 3 were recoded to a 2. This approach was taken to align the data with the score algorithm.^31,32^

An important assumption of IRT, unidimensionality, is that 1 unobserved construct (θ) is responsible for observed item responses.^33^ To address this assumption, we ran confirmatory IRT to understand whether SA and RRB should be evaluated separately (across 2 factors) or together (a single, unidimensional factor). Models were assessed using several goodness-of-fit indices, including the comparative fit index, the Tucker-Lewis index, the root mean square error of approximation, M2/C2, and the standardized root mean square residual. Comparative fit index and Tucker-Lewis index values greater than 0.92 indicate a good fit.^35,36^ Root mean square error of approximation and standardized root mean square residual values of less than 0.06 are considered excellent, and the M2/C2 is interpreted similar to a χ^2^ value.^35,36,37^

#### Differential Item Functioning

The IRT framework assumes all test items are invariant across subpopulations.^38^ For example, we assume both male children and female children as well as White and Black/African American children have the same ADOS-2 item response profiles defined by *a_i_* and *b_i_* parameters. Differential item functioning (DIF) is a statistical approach to address this assumption.^39^ Specifically, DIF is used to evaluate the extent to which an item may be performing in an unexpected manner or measuring different abilities (across *a_i_* or *b_i_*) across subgroups. Ultimately, DIF is one approach to detecting measurement inequities or biases across groups.^40^

There are 2 types of DIF: uniform and nonuniform. Uniform DIF is when *b_i_* is different across populations. This reflects a scenario wherein 1 group has a systematically higher or lower probability of item response across all levels of ASD severity. Thus, uniform DIF is consistent with notions of systematic bias in item responses. Nonuniform DIF describes a situation where *a_i_* is different depending on levels of θ; this type of DIF is analogous to differences in amounts of measurement error between groups.^41^ Item response characteristic curves (ICCs) are a useful tool to visualize DIF because they graph the probability of response on the *y*-axis against latent trait levels on the *x*-axis. Differences in ICCs along the *x*-axis demarcate differences in *b_i_*. The steepness or flatness in ICCs reflects differences in *a_i_*

Statistically, likelihood ratio χ^2^ tests, from the graded response IRT model, were used to identify presence of each DIF. However, small differences in DIF can lead to a positive χ^2^ test in large samples. Thus, *R*^2^, regression coefficients, and expected standardized score difference (ESSD) were used to assess item-level magnitude of DIF. A cutoff of 0.02 was used for *R*^2^ values,^42,43^ and a 10% change in regression coefficients (∆β) was indicative of a meaningful association.^44^ ESSD can be interpreted using Cohen guidelines for estimated effect sizes.^43^ The overall estimated effect of all the items on expected scores, or differential test functioning (DTF), was measured using unsigned expected test score difference in the sample (UETSDS) and expected test standardized score difference (ETSSD). An ETSSD plus or minus 0.2 is considered a meaningful change. We also consider an UETSDS of greater than 2, which is interpreted in terms of total scale points (ie, the ADOS-2 score), as meaningful change. Two-sided *P* < .05 was considered significant. Analyses were conducted using Stata statistical software version 15.0 (StataCorp) and R packages lavaan, psych, mirt, and lordif in R statistical software version 4.1.3 (R Project for Statistical Computing).^45,46,47,48^ Overall, there were few missing data (<1%). The models used complete case analysis. Data were analyzed from July 2021 to February 2022.

## Results

### Participants

The analytical sample consisted of 6269 unique children (1619 Black/African American children [25.9%]; 3151 White children [50.3%]; 4970 male children [79.4%]). Participants ranged in age from 1.7 to 17.9 years (mean [SD] age, 6.77 [3.27] years). See Table 1 for demographic characteristics of the sample. Descriptive statistics for ADOS-2 item scores and classifications, by race and sex, are shown in Table 2. Item-level scores across algorithms, which are stratified by race and sex, are shown in eTable 1 and eTable 2 in the Supplement. Sociodemographic differences are not statistically evaluated in these tables because DIF testing is the appropriate format for understanding group differences.

**Table 2.** ADOS-2 Item Scores by Race and Sex

Item	Score, mean (SD)			
	Race		Sex	
	White	Black/African American	Female	Male
Children, No. (%)	3147 (50.3)	1618 (25.9)	1292 (20.6)	4963 (79.4)
ADOS-2 items				
Eye contact	1.19 (0.98)	1.35 (0.94)	1.20 (0.98)	1.26 (0.97)
Gaze^a^	0.78 (0.75)	0.96 (0.77)	0.86 (0.77)	0.88 (0.77)
Facial expressions	0.69 (0.66)	0.81 (0.67)	0.74 (0.69)	0.75 (0.66)
Vocalization	0.79 (0.74)	0.97 (0.78)	0.85 (0.78)	0.87 (0.76)
Shared enjoyment	0.58 (0.74)	0.68 (0.77)	0.61 (0.76)	0.62 (0.75)
Social overtures	0.88 (0.65)	1.00 (0.69)	0.93 (0.70)	0.95 (0.67)
Responding to joint attention	0.80 (0.73)	0.96 (0.77)	0.83 (0.75)	0.87 (0.75)
Gestures	0.63 (0.71)	0.85 (0.75)	0.70 (0.74)	0.73 (0.74)
Social response	0.89 (0.73)	1.04 (0.79)	0.95 (0.78)	0.97 (0.76)
Initiation of joint attention	0.69 (0.77)	0.86 (0.81)	0.73 (0.81)	0.77 (0.79)
Stereotyped language	0.63 (0.73)	0.72 (0.80)	0.68 (0.78)	0.68 (0.76)
Sensory interest	0.49 (0.77)	0.67 (0.85)	0.51 (0.78)	0.59 (0.82)
Repetitive interest	0.51 (0.82)	0.57 (0.85)	0.53 (0.83)	0.56 (0.84)
Hand mannerisms	0.91 (0.84)	0.98 (0.83)	0.82 (0.82)	1.01 (0.83)
ADOS-2 CSS				
CSS	5.16 (2.92)	5.71 (2.82)	5.15 (2.99)	5.48 (2.88)
Social affect CSS	5.27 (2.79)	5.77 (2.71)	5.30 (2.84)	5.51 (2.77)
Restrictive, repetitive behaviors CSS	5.67 (3.10)	5.97 (3.04)	5.45 (3.12)	5.98 (3.03)
ADOS-2 status, children, No. (%)				
No ASD/autism	1139 (36.2)	443 (27.4)	472 (36.5)	1561 (31.5)
ASD	393 (12.5)	195 (12.1)	144 (11.1)	599 (12.1)
Autism	1614 (51.3)	980 (60.6)	676 (52.3)	2803 (56.5)

Abbreviations: ADOS-2, Autism Diagnostic Observation Schedule, Second Edition; ASD, autism spectrum disorder; CSS, Calibrated Severity Score.

^a^ All items with 3 categories were collapsed to 2 categories, except for gaze, which is dichotomous.

### Dimensionality

The fit statistics comparing the unidimensional and 2-factor confirmatory factor analysis models are shown in eTable 3 in the Supplement. The unidimensional model was superior to the 2-factor model across all modules and algorithms for each of the fit indices. The unidimensional model was also superior to the SA factor, whereas the RRB factor appeared to be a good fit. Therefore, all IRT-based analyses analyzed SA and RRB as a single domain of ASD (unidimensional).

### Differential Item Functioning

Each of the 10 SA and 4 RRB items was evaluated for DIF across race and sex for each of the 5 algorithms. Only items that were significant according to the χ^2^ DIF tests are shown in Table 3 and Table 4. A total of 140 item-level DIF analyses were performed, and only 16 items (11%) were significant.

**Table 3.** Item Response Theory Parameters for Items With Suspected DIF by Race

Module, construct, and item	*a*	*b*_1_	*b*_2_	DIF type	*R* ^2^	∆β	ESSD	ETSSD^a^	UETSDS^a^
Module 1.1, SA, gaze^b^									
White	2.60	−1.61	−0.18	Uniform	0.012	0.02	0.08	0.05	0.91
Black/African American	2.24	−1.55	0.12						
Module 1.2, SA, shared enjoyment^c^									
White	2.01	−0.07	1.14	Uniform	0.01	0.03	0.26	0.008	0.41
Black/African American	1.79	0.07	1.56						
Module 2.2, RRB, repetitive interests^d^									
White	0.54	0.69	1.86	Nonuniform	0.01	0.02	1.22	0.03	0.34
Black/African American	0.63	1.80	2.66						
Module 3, SA, facial expressions									
White	1.72	0.05	2.00	Nonuniform	0.01	0.002	0.11	0.04	0.25
Black/African American	1.32	−0.12	2.15						
Module 3, SA, quality of overtures									
White	2.58	−0.51	1.63	Uniform	0.01	0.03	−0.20	NA	NA
Black/African American	2.47	−0.38	1.95						
Module 3, SA, showing									
White	1.04	0.77	3.29	Uniform	0.01	0.01	0.22	NA	NA
Black/African American	1.11	0.49	2.86						
Module 3, SA, initiation of joint attention									
White	1.10	0.15	2.40	Uniform	0.002	0.005	0.22	NA	NA
Black/African American	1.08	−0.13	2.32						
Module 3, RRB, stereotyped language									
White	0.86	0.26	3.02	Nonuniform	0.001	0.02	0.22	NA	NA
Black/African American	0.57	0.76	4.55						

Abbreviations: *a,* item discrimination; *b,* item difficulty for each level of response; DIF, differential item functioning; ESSD, expected standardized score difference; ETSSD, expected test standardized score difference; NA, not applicable; RRB, repetitive, restrictive behavior; SA, social affect; UETSDS, unsigned expected test score difference in the sample.

^a^ ETSSD and UETSDS are test-level statistics that assess the effect of differential functioning of all items on the total score.

^b^ Module 1.1 refers to module 1, no words.

^c^ Module 1.2 refers to module 1, words.

^d^ Module 2.1 refers to module 2, <5 years; module 2.2 refers to module 2, >5 years.

**Table 4.** Item Response Theory Parameters for Items With Suspected DIF by Sex

Module, construct, and item	*a*	*b*_1_	*b*_2_	DIF type	*R* ^2^	∆β	ESSD	ETSSD	UETSDS
Module 1.1, RRB, hand mannerisms									
Male	0.91	−3.03	−1.03	Nonuniform	0.01	0.01	−0.45	0.01	0.18
Female	1.15	−1.82	−0.41						
Module 1.2, SA, facial expressions									
Male	2.31	−0.89	1.43	Nonuniform	0.01	0.01	0.03	<0.001	0.07
Female	1.93	−1.42	1.37						
Module 2.1, SA, unusual eye contact									
Male	1.51	−0.42		Nonuniform	0.001	0.001	−0.03	−0.12	0.61
Female	2.77	−0.38							
Module 2.1, RRB, hand mannerisms									
Male	1.18	−1.06	1.18	Nonuniform	0.03	0.001	−0.66	NA	NA
Female	0.94	−0.67	0.94						
Module 2.2, RRB, hand mannerisms									
Male	2.23	−0.84	0.32	Nonuniform	0.01	0.01	−0.64	−0.10	0.59
Female	1.94	−0.61	0.73						
Module 3, SA, gaze									
Male	3.53	0.00	1.34	Uniform	0.003	0.001	0.15	−0.04	0.20
Female	3.04	−0.12	1.34						
Module 3, SA, initiation of joint attention									
Male	1.13	−0.01	2.22	Uniform	0.002	0.006	−0.26	NA	NA
Female	0.95	0.33	2.85						
Module 3, RRB, hand mannerisms									
Male	0.87	0.12	2.00	Uniform	0.02	0.04	−0.55	NA	NA
Female	0.98	0.55	2.46						

Abbreviations: *a,* item discrimination; *b,* item difficulty for each level of response; DIF, differential item functioning; ESSD, expected standardized score difference; ETSSD, expected test standardized score difference; NA, not applicable; RRB, repetitive, restrictive behaviors; SA, social affect; UETSDS, unsigned expected test score difference in the sample.

### Race

Item-level DIF by race is shown in Table 3. Eight items had significant DIF (2 items for module 1, 1 item for module 2, and 5 items for module 3). More than one-half of the items with DIF (6 of 8) involved SA. No item had DIF consistently across all modules. In terms of item discrimination (*a*), 6 of 8 items had poorer discrimination among Black/African American children compared with White children. Most items (5 of 8) had uniform DIF with higher difficulty, or greater *b_i_* values, in Black/African American children compared with White children. The overall magnitude of DIF and DTF was small. This can be seen in the low *R*^2^ (0.001-0.012), β (0.002-0.03), ETSSD (0.008-0.05), and UETSDS (<1 point) values. However, ESSD was large for repetitive interests (1.22; module 2.2).

### Sex

Item-level DIF by sex is shown in Table 4. Five unique items had significant DIF (2 items for module 1, 2 items for module 2, and 3 items for module 3). DIF was equally split between SA and RRB, and poorer discrimination (3 of 4 items) was the most consistent pattern. A little more than one-half (5 of 8 items) of DIF was nonuniform with poorer discrimination, for female children compared with male children. Items had higher difficulty half of the time in female children compared with male children. Hand mannerisms demonstrated DIF across all 3 modules, with estimated effect sizes in the moderate range (−0.45 to −0.64) and *R*^2 ^> 0.02. Magnitude of DIF and DTF was small for all other items (*R*^2^, 0.003 to 0.01; β, 0.001 to 0.04; ETSSD, 0.001 to 0.1; and UETSDS, <1 point). See eFigure 1 and eFigure 2 in the Supplement for visualization of ICCs for each item with DIF.

## Discussion

Measurement has been a key focus of discussion in the debate about what has driven historical ASD diagnostic disparities across sex and race. Cogent arguments have been put forth about limitations in the diagnostic nosology and the limited inclusivity of the phenotype in the standardization samples used to psychometrically evaluate reference standard measures such as the ADOS-2.^49^ This bias could result in underdetection among minoritized racial groups and female children. Surprisingly, to our knowledge, only 2 studies^16,29^ have used modern measurement methods (eg, IRT) to examine item-level biases on the ADOS.

Consistent with prior work, the findings of this cross-sectional study suggest minimal overall item-level bias of the ADOS-2.^16,29^ A total of 140 item-level DIF analyses were performed. Of these analyses, the χ^2^ test, which is highly sensitive owing to the large sample size, was significant for only 11% of items. Of the 16 significant items, estimated effect sizes were moderate to large for 2 RRB items (repetitive interests and hand mannerisms). The impact of these 2 items on the overall ADOS-2 algorithms, as measured by DTF indices, was small.

When comparing ADOS-2 DIF for Black/African American children compared with White children, minimal DIF was observed. When DIF did occur, estimated effect sizes were small for all items but repetitive interests. There are 2 patterns worth considering, however. First, when DIF was present, it was most frequently observed in the SA domain. Second, the direction of bias was generally greater difficulty, resulting in underestimation of ASD severity for Black/African American children. Discrimination was poorer as well, suggesting these items do not detect ASD as effectively in Black/African American children. This finding sits somewhat in contrast to Harrison et al^16^ who reported overestimation of scores for Black/African American children; however, only 3 items were identified with DIF,^16^ of which only 1 was in the diagnostic algorithm (not repetitive interests). All items evaluated in the present study are included in the diagnostic algorithm, which has direct implications for diagnostic bias.

We are unaware of any data supporting biological mechanisms that could give rise to phenotypic differences of ASD related to race. This is likely because race is a social, rather than biological, construct. Nevertheless, the literature is mixed in terms of phenotypic differences between these groups. For instance, Sell et al^50^ and Tek et al^51^ found differences in core ASD symptoms between racial groups; however, Cuccaro et al,^52^ Fombonne et al,^53^ and Stronach et al^54^ did not. If racial differences are found, we believe they are likely a product of differential referral trends or study selection biases. For instance, Black/African American children who are seen clinically may be phenotypically different from White children as the result of being referred for more general developmental symptoms that may be less specific to ASD,^55,56^ experiencing greater delays due to challenges accessing high-quality services,^57,58^ having lower socioeconomic status secondary to structural racism,^59,60^ and cultural factors, particularly those related to identification of SA.^61^

A different pattern of DIF emerged for sex. Sex-related DIF was equally split between RRB and SA. However, RRB-related DIF was solely confined to hand mannerisms, which demonstrated bias across all modules and algorithms. The estimated effect sizes for this item were moderate, with generally greater difficulty. SA, on the other hand, was split across 4 separate items across modules. Although DIF was nonuniform, poorer discrimination (3 of 4 items) was the most consistent pattern. These findings raise direct concerns about the hand mannerisms item. Given the brevity of the diagnostic algorithm for RRB, which only includes 4 items, having 25% of the items consistently underestimate ASD in female children is notable and worth prompting further research.

This finding is somewhat consistent with the literature of underdetection. For instance, Lai et al^62^ found that 20% of female adults with ASD met ADOS criteria, compared with 58% of male children, and Ratto et al^63^ discovered that female children with higher intelligence quotient scores were significantly less likely to meet on the Autism Diagnostic Interview–Revised. Most of the literature discussing underdetection has focused on SA, particularly in association with camouflaging ASD symptoms.^64^ Our study suggests that there is a greater number of items at risk for bias in the ADOS-2 related to SA. However, the findings were inconsistent (in terms of items), and estimated effect sizes were small.

### Limitations and Strengths

This study’s findings should be considered in light of its weaknesses and strengths. For limitations, the study was single site, we were unable to investigate bias in other racial or ethnic groups (because of the small sample sizes), and there was a lack of information on intellectual/adaptive functioning and clinical diagnoses. Another notable limitation was the information on ethnicity. We attempted to address potential confounding of ethnicity, in the racial analysis, by removing those who were Hispanic. However, the lack of information on ethnicity did not permit full exclusion of this group. Furthermore, not all clinicians were ADOS-2 research reliable, although they were all trained and monitored by research reliable administrators. For strengths, this study fills a critical gap in the literature, the sample was large and heterogenous, and the statistical methods were advanced.

## Conclusions

In summary, our findings suggest minimal DIF of the ADOS-2. When DIF did occur, 2 differential patterns of measurement bias occurred across race and sex. For Black/African American children, DIF was most frequently observed in the SA domain with a pattern of greater difficulty and poorer discrimination. Importantly, estimated effect sizes were small for all items except repetitive interests. For sex, the hand mannerisms item demonstrated consistent bias across ADOS-2 modules among female children compared with male children. At the macro level, these findings are consistent with Harrison et al,^16^ since their study suggests the magnitude of the bias was small and likely to have little epidemiological impact. At the individual level, the DIF observed for Black/African American and female children could result in underestimation or underdetection of ASD. Our findings call for replication using multisite samples across a wide range of racial, ethnic, and sex groups.

## Notes

Supplement. **eTable 1.** IRT Parameters and ADOS Characteristics for Items With Suspected DIF by Sex **eTable 2.** IRT Parameters and ADOS Characteristics for Items With Suspected DIF by Race **eTable 3.** Fit Statistics by ADOS-2 Algorithm **eFigure 1.** Item Response Theory Curves by Race **eFigure 2.** Item Response Theory Curves by Sex
