# Supplementary material for: Analysis of Race and Sex Bias in the Autism Diagnostic Observation Schedule (ADOS-2)
Source: JAMA Netw Open. 2022 Apr 26;5(4):e229498. doi: 10.1001/jamanetworkopen.2022.9498 (PMC9044110; doi:10.1001/jamanetworkopen.2022.9498)
Supplement: Supplement. — eTable 1. IRT Parameters and ADOS Characteristics for Items With Suspected DIF by Sex eTable 2. IRT Parameters and ADOS Characteristics for Items With Suspected DIF by Race eTable 3. Fit Statistics by ADOS-2 Algorithm eFigure 1. Item Response Theory Curves by Race eFigure 2. Item Response Theory Curves by Sex [file jamanetwopen-e229498-s001.pdf]

Supplemental Online Content

Kalb LG, Singh V, Hong JS, et al. Analysis of race and sex bias in the Autism Diagnostic Observation Schedule (ADOS-2). *JAMA Netw Open*. 2022;5(4):e229498. doi:10.1001/jamanetworkopen.2022.9498

eTable 1. IRT Parameters and ADOS Characteristics for Items With Suspected DIF by Sex
eTable 2. IRT Parameters and ADOS Characteristics for Items With Suspected DIF by Race
eTable 3. Fit Statistics by ADOS-2 Algorithm
eFigure 1. Item Response Theory Curves by Race
eFigure 2. Item Response Theory Curves by Sex

This supplemental material has been provided by the authors to give readers additional information about their work.

© 2022 Kalb LG et al. *JAMA Network Open.*

eTable 1: IRT Parameters and ADOS Characteristics for Items With Suspected DIF by Sex

	Mod 1 - Algorithm 1		Mod 1 - Algorithm 2		Mod 2 - Algorithm 1		Mod 2 - Algorithm 2		Mod3	
Sex	F	M	F	M	F	M	F	M	F	M
N (%)	184 (22.7%)	624 (77.3%)	198 (19.1%)	841 (80.9%)	189 (22.8%)	638 (77.2%)	110 (18.9%)	472 (81.1%)	611 (20.4%)	2390 (79.6%)
ADOS-2 Items (M, SD)										
Eye Contact	1.71 (0.71)	1.75 (0.66)	1.62 (0.79)	1.45 (0.89)	1.17 (0.99)	1.23 (0.97)	1.24 (0.98)	1.33 (0.94)	0.92 (1.00)	1.06 (1.00)
Gaze	1.46 (0.68)	1.42 (0.67)	0.96 (0.70)	0.89 (0.67)	0.93 (0.79)	1.00 (0.83)	1.15 (0.81)	1.20 (0.81)	0.57 (0.66)	0.63 (0.70)
Facial Expressions	1.32 (0.62)	1.24 (0.60)	1.05 (0.57)	0.87 (0.59)	0.53 (0.58)	0.65 (0.59)	0.81 (0.67)	0.74 (0.64)	0.51 (0.63)	0.60 (0.65)
Vocalization	1.73 (0.56)	1.68 (0.58)	0.94 (0.70)	0.87 (0.69)	0.62 (0.72)	0.66 (0.70)	0.90 (0.75)	0.98 (0.79)	0.61 (0.67)	0.69 (0.70)
Shared Enjoyment	1.24 (0.79)	1.07 (0.77)	0.82 (0.75)	0.64 (0.73)	0.35 (0.64)	0.38 (0.66)	0.57 (0.76)	0.57 (0.73)	0.44 (0.68)	0.56 (0.72)
Social Overtures	1.61 (0.61)	1.52 (0.61)	1.18 (0.67)	1.08 (0.64)	0.79 (0.63)	0.84 (0.60)	0.98 (0.68)	1.06 (0.67)	0.68 (0.59)	0.75 (0.61)
RJA	1.20 (0.84)	1.14 (0.81)	1.13 (0.79)	1.06 (0.81)	0.69 (0.64)	0.67 (0.69)	0.80 (0.71)	0.74 (0.68)	0.67 (0.67)	0.81 (0.72)
Gestures	1.22 (0.71)	1.25 (0.69)	0.95 (0.72)	0.91 (0.72)	0.89 (0.77)	0.94 (0.75)	0.94 (0.76)	1.02 (0.73)	0.35 (0.56)	0.42 (0.60)
Social Response	1.72 (0.61)	1.71 (0.61)	1.29 (0.82)	1.21 (0.82)	0.62 (0.75)	0.62 (0.74)	0.84 (0.82)	0.80 (0.82)	0.72 (0.59)	0.81 (0.61)
IJA	1.44 (0.83)	1.42 (0.79)	1.02 (0.88)	0.97 (0.86)	0.54 (0.72)	0.52 (0.70)	0.72 (0.77)	0.68 (0.78)	0.48 (0.63)	0.62 (0.68)
Stereotyped Language	0.98 (0.95)	0.82 (0.92)	0.95 (0.82)	0.87 (0.82)	0.72 (0.77)	0.69 (0.72)	0.88 (0.83)	0.79 (0.82)	0.46 (0.62)	0.54 (0.66)
Sensory Interest	1.18 (0.90)	1.28 (0.86)	0.88 (0.86)	0.93 (0.87)	0.44 (0.72)	0.62 (0.81)	0.62 (0.83)	0.79 (0.87)	0.18 (0.49)	0.24 (0.56)
Repetitive Interest	1.17 (0.95)	1.25 (0.91)	0.93 (0.93)	0.92 (0.92)	0.42 (0.75)	0.55 (0.81)	0.47 (0.77)	0.57 (0.85)	0.25 (0.60)	0.25 (0.60)
Hand Mannerisms	1.46 (0.75)	1.60 (0.64)	1.32 (0.75)	1.41 (0.72)	0.85 (0.78)	1.11 (0.80)	0.92 (0.78)	1.16 (0.79)	0.45 (0.66)	0.66 (0.77)
ADOS-2 CSS (M, SD)										
CSS	6.88 (2.44)	6.73 (2.28)	6.43 (2.70)	6.13 (2.65)	4.80 (2.99)	5.24 (2.75)	5.39 (2.73)	5.65 (2.63)	4.28 (2.91)	4.95 (3.02)
SA CSS	6.51 (2.42)	6.26 (2.26)	6.08 (2.65)	5.65 (2.60)	5.10 (2.85)	5.28 (2.68)	5.64 (2.73)	5.74 (2.61)	4.69 (2.86)	5.29 (2.95)
RRB CSS	7.60 (2.26)	7.90 (2.04)	7.37 (2.35)	7.51 (2.37)	5.35 (2.76)	6.01 (2.60)	5.61 (2.82)	6.13 (2.71)	4.19 (3.04)	4.92 (3.13)
ADOS-2 Status (N, %)										
No ASD/Autism	22 (12.0%)	58 (9.29%)	35 (17.7%)	163 (19.4%)	75 (39.7%)	195 (30.6%)	35 (31.8%)	134 (28.5%)	305 (49.9%)	1011 (42.3%)
ASD	12 (6.52%)	78 (12.5%)	24 (12.1%)	120 (14.3%)	28 (14.8%)	107 (16.8%)	7 (6.36%)	29 (6.16%)	73 (11.9%)	265 (11.1%)
Autism	150 (81.5%)	488 (78.2%)	139 (70.2%)	558 (66.3%)	86 (45.5%)	336 (52.7%)	68 (61.8%)	308 (65.4%)	233 (38.1%)	1113 (46.6%)

*M (Male), F (Female), SA (Social Affect), RRB (Repetitive, Restrictive Behaviors), RAJ (Response to Joint Attention), IJA (Initiates Joint Attention)

© 2022 Kalb LG et al. *JAMA Network Open.*

eTable 2: IRT Parameters and ADOS Characteristics for Items With Suspected DIF by Race

	Mod 1 - Algorithm 1		Mod 1 - Algorithm 2		Mod 2 - Algorithm 1		Mod 2 - Algorithm 2		Mod3	
Race	White	Black/AA	White	Black/AA	White	Black/AA	White	Black/AA	White	Black/AA
N (%)	287(50.0%)	287 (50.0%)	417 (59.4%)	285 (40.6%)	439 (71.4%)	176 (28.6%)	209 (50.1%)	206 (49.9%)	1765 (72.3%)	657 (27.7%)
ADOS-2 Items (M, SD)										
Eye Contact	1.76 (0.66)	1.78 (0.63)	1.44 (0.90)	1.52 (0.85)	1.24 (0.97)	1.14 (0.99)	1.26 (0.97)	1.34 (0.94)	1.01 (1.00)	1.13 (0.99)
Gaze	1.46 (0.68)	1.38 (0.66)	0.83 (0.69)	0.96 (0.67)	0.92 (0.82)	0.99 (0.82)	1.12 (0.81)	1.22 (0.81)	0.58 (0.67)	0.69 (0.72)
Facial Expressions	1.28 (0.60)	1.26 (0.60)	0.88 (0.60)	0.90 (0.58)	0.66 (0.61)	0.58 (0.57)	0.78 (0.66)	0.76 (0.65)	0.55 (0.63)	0.66 (0.67)
Vocalization	1.66 (0.59)	1.74 (0.52)	0.80 (0.67)	0.94 (0.69)	0.64 (0.70)	0.73 (0.71)	0.94 (0.79)	1.02 (0.78)	0.67 (0.68)	0.70 (0.70)
Shared Enjoyment	1.13 (0.76)	1.09 (0.77)	0.68 (0.76)	0.65 (0.71)	0.40 (0.68)	0.40 (0.67)	0.61 (0.77)	0.57 (0.73)	0.50 (0.69)	0.61 (0.76)
Social Overtures	1.51 (0.62)	1.57 (0.59)	1.02 (0.65)	1.15 (0.63)	0.81 (0.60)	0.87 (0.62)	1.04 (0.67)	1.03 (0.68)	0.74 (0.60)	0.72 (0.60)
RJA	1.13 (0.80)	1.25 (0.79)	0.98 (0.79)	1.17 (0.78)	0.64 (0.66)	0.71 (0.69)	0.68 (0.68)	0.78 (0.69)	0.75 (0.70)	0.87 (0.74)
Gestures	1.18 (0.73)	1.29 (0.64)	0.85 (0.68)	1.00 (0.74)	0.90 (0.74)	1.02 (0.77)	0.99 (0.76)	1.04 (0.72)	0.38 (0.58)	0.49 (0.63)
Social Response	1.72 (0.58)	1.72 (0.59)	1.17 (0.84)	1.30 (0.81)	0.63 (0.75)	0.60 (0.76)	0.78 (0.82)	0.90 (0.83)	0.78 (0.59)	0.80 (0.62)
IJA	1.41 (0.81)	1.44 (0.77)	0.93 (0.86)	0.99 (0.86)	0.53 (0.71)	0.51 (0.73)	0.63 (0.77)	0.73 (0.78)	0.55 (0.66)	0.67 (0.68)
Stereotyped Language	0.84 (0.92)	0.92 (0.94)	0.80 (0.81)	0.93 (0.81)	0.68 (0.71)	0.71 (0.77)	0.81 (0.83)	0.85 (0.85)	0.53 (0.65)	0.49 (0.64)
Sensory Interest	1.24 (0.89)	1.29 (0.84)	0.87 (0.87)	0.97 (0.89)	0.58 (0.78)	0.57 (0.81)	0.77 (0.87)	0.80 (0.87)	0.22 (0.54)	0.25 (0.57)
Repetitive Interest	1.30 (0.90)	1.17 (0.95)	0.93 (0.92)	0.91 (0.92)	0.53 (0.80)	0.51 (0.79)	0.69 (0.88)	0.43 (0.77)	0.26 (0.62)	0.22 (0.55)
Hand Mannerisms	1.56 (0.68)	1.57 (0.69)	1.36 (0.74)	1.38 (0.71)	1.08 (0.81)	0.97 (0.82)	1.10 (0.83)	1.10 (0.78)	0.63 (0.76)	0.52 (0.69)
ADOS-2 CSS (M, SD)										
CSS	6.73 (2.30)	6.84 (2.17)	5.90 (2.81)	6.40 (2.50)	5.10 (2.86)	5.18 (2.78)	5.52 (2.78)	5.73 (2.61)	4.70 (2.94)	5.05 (3.04)
SA CSS	6.27 (2.32)	6.40 (2.14)	5.46 (2.63)	5.96 (2.53)	5.20 (2.75)	5.28 (2.63)	5.57 (2.73)	5.83 (2.64)	5.03 (2.87)	5.51 (2.99)
RRB CSS	7.86 (2.09)	7.90 (2.05)	7.37 (2.51)	7.54 (2.25)	5.90 (2.65)	5.70 (2.69)	6.14 (2.82)	6.00 (2.64)	4.80 (3.14)	4.51 (3.10)
ADOS-2 Status (N, %)										
No ASD/Autism	30 (10.5%)	20 (6.97%)	96 (23.0%)	44 (15.4%)	152 (34.6%)	55 (31.2%)	65 (31.2%)	55 (26.7%)	786 (44.6%)	268 (40.8%)
ASD	31 (10.8%)	37 (12.9%)	67 (16.1%)	36 (12.6%)	66 (15.0%)	35 (19.9%)	12 (5.77%)	16 (7.77%)	216 (12.2%)	71 (10.8%)
Autism	226 (78.7%)	230 (80.1%)	254 (60.9%)	205 (71.9%)	221 (50.3%)	86 (48.9%)	131 (63.0%)	135 (65.5%)	762 (43.2%)	318 (48.4%)

*M (Male), F (Female), SA (Social Affect), RRB (Repetitive, Restrictive Behaviors), RAJ (Response to Joint Attention), IJA (Initiates Joint Attention)

© 2022 Kalb LG et al. *JAMA Network Open.*

eTable 3: Fit Statistics by ADOS-2 Algorithm

Module	Model	M2/C2	RMSEA	SRMR	TLI	CFI
Module 1.1	Two Factor	691.715	0.109 (0.102-0.117)	0.19	0.89	0.90
	Unidimensional	278.699	0.064 (0.057-0.072)	0.06	0.96	0.97
	RRB Factor	7.234	0.057 (0.016-0.104)	0.03	0.92	0.97
	SA Factor	392.108	0.129 (0.118-0.141)	0.19	0.91	0.93
Module 1.2	Two Factor	768.831	0.102 (0.096-0.109)	0.20	0.91	0.93
	Unidimensional	329.332	0.063 (0.057-0.07)	0.05	0.97	0.97
	RRB Factor	8.043	0.054 (0.019-0.095)	0.03	0.95	0.98
	SA Factor	495.219	0.13 (0.12-0.14)	0.20	0.92	0.94
Module 2.1	Two Factor	856.196	0.121 (0.114-0.129)	0.20	0.85	0.87
	Unidimensional	500.909	0.091 (0.084-0.098)	0.06	0.91	0.93
	RRB Factor	0.268	0 (0-0.034)	0.01	1.01	1.00
	SA Factor	457.576	0.139 (0.128-0.15)	0.16	0.85	0.89
Module 2.2	Two Factor	609.028	0.12 (0.111-0.129)	0.21	0.87	0.89
	Unidimensional	472.017	0.105 (0.096-0.114)	0.07	0.90	0.92
	RRB Factor	15.274	0.107 (0.061-0.16)	0.05	0.92	0.97
	SA Factor	301.433	0.132 (0.119-0.146)	0.18	0.89	0.92
Module 3	Two Factor	2094.974	0.102 (0.098-0.106)	0.18	0.91	0.92
	Unidimensional	1463.464	0.085 (0.082-0.089)	0.06	0.94	0.95
	RRB Factor	30.919	0.069 (0.049-0.092)	0.04	0.91	0.97
	SA Factor	1256.446	0.123 (0.117-0.129)	0.15	0.92	0.94

*RMSEA (Root Mean Squared Error of Association), TLI (Tucker Lewis Index), CFI (Comparative Fit Index)

© 2022 Kalb LG et al. *JAMA Network Open.*

eFigure 1: Item Response Theory Curves by Race

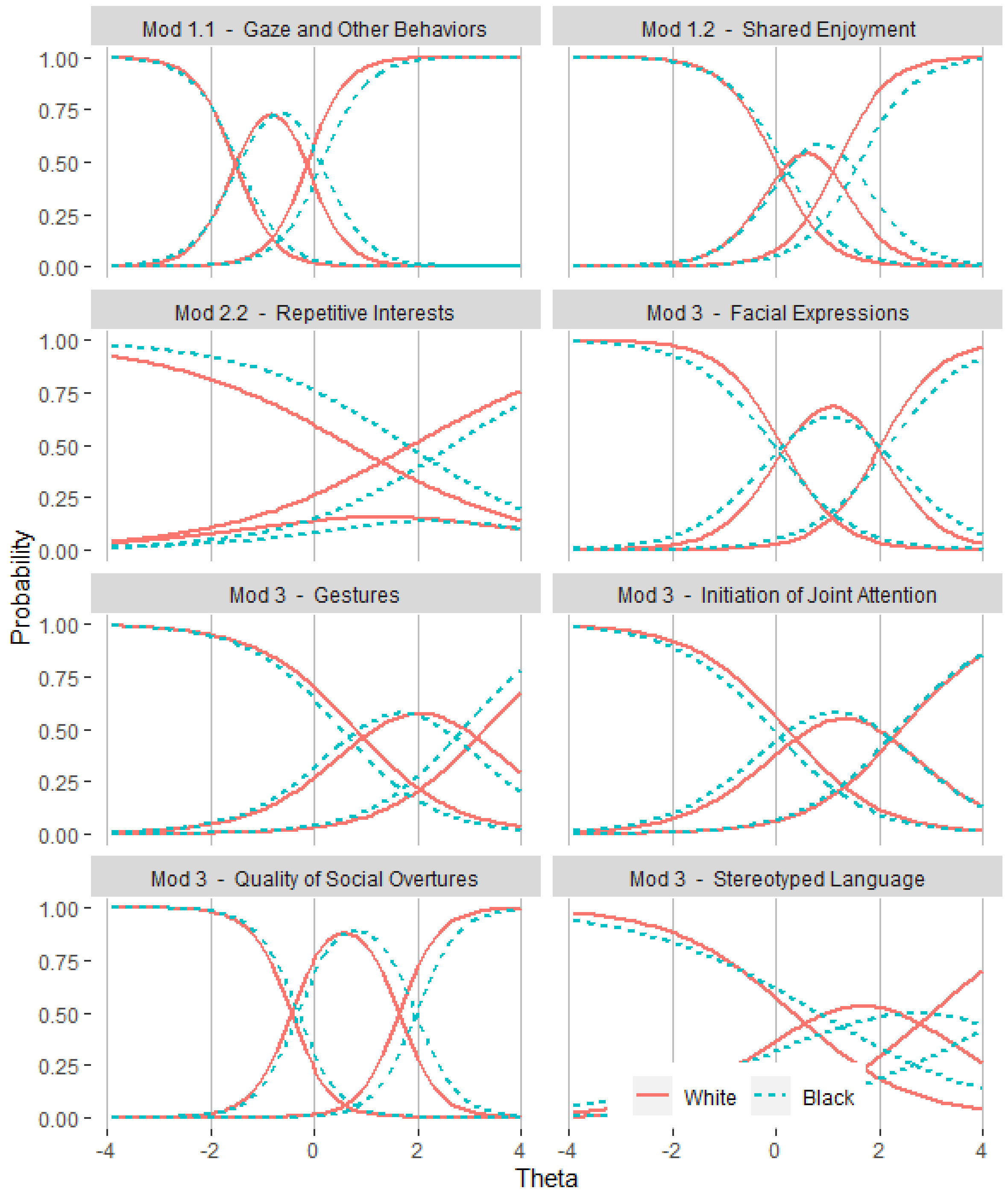

© 2022 Kalb LG et al. *JAMA Network Open.*

eFigure 2: Item Response Theory Curves by Sex

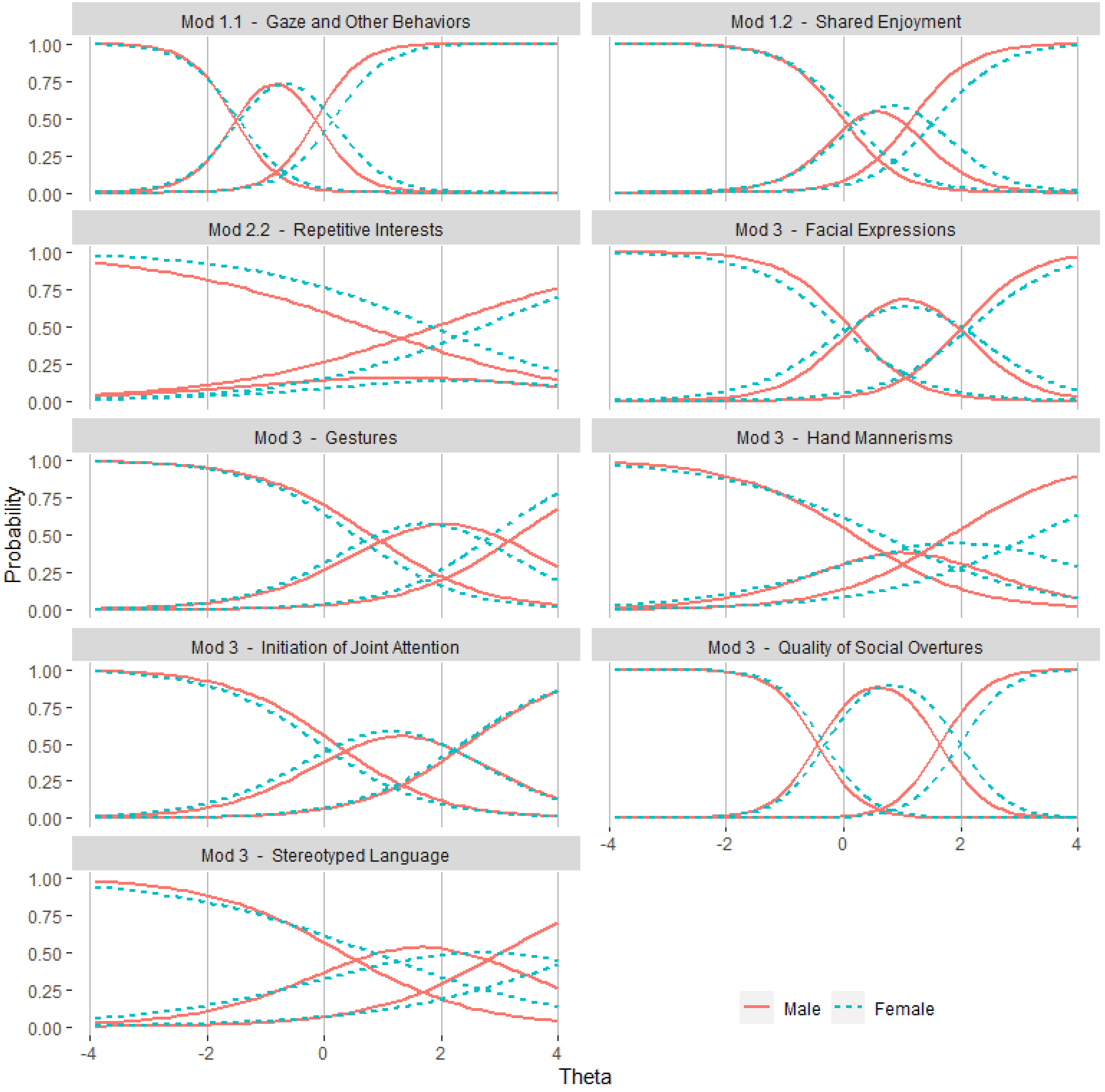

© 2022 Kalb LG et al. *JAMA Network Open.*
